# Effect of bisphosphonate on temporomandibular joint in osteopenia-induced rats by botulinum toxin A injection on masticatory muscle: a preliminary study

**DOI:** 10.1186/s40902-019-0193-5

**Published:** 2019-03-04

**Authors:** Jae-Young Kim, Dae-Hoon Kim, Hyo-Won Jang, Kwang-Ho Park, Jong-Ki Huh

**Affiliations:** Department of Oral and Maxillofacial Surgery, Gangnam Severance Hospital, Yonsei University College of Dentistry, 211 Eonju-ro, Gangnam-gu, Seoul, 06273 South Korea

**Keywords:** Bisphosphonate, Botulinum toxin, Mandibular condyle, Temporomandibular joint

## Abstract

**Background:**

Botulinum toxin injection on the masticatory muscle induces the osteopenic condition on the ipsilateral condyle. Bisphosphonate suppresses bone resorption and is used to treat osteopenic or osteoporotic condition. This study aimed to evaluate the effect of bisphosphonate administration on prevention of condylar resorption and botulinum toxin A-induced disuse osteopenia in rats.

**Results:**

The volume of the condyle and bone volume/tissue volume (BV/TV, %) showed a strong tendency towards statistical significance (*p* = 0.052 and 0.058). Trabecular thickness (Tb.Th, mm) and trabecular number (Tb.N, 1/mm) were significantly smaller in the Botox group than in the other groups (*p* < 0.05). The volume of the condyle and BV/TV in the bisphosphonate 100 and bisphosphonate 200 groups showed similar values when compared with the control group.

**Conclusion:**

Bisphosphonate administration after botulinum toxin A injection in the masticatory muscles appears to prevent condyle resorption and botulinum toxin-induced disuse osteopenia in rats.

## Background

Botulinum toxin causes transient paralysis of muscles by blocking acetylcholine release at the neuromuscular junction [1]. Botulinum toxin A (BTX-A) is most widely used among the seven types of toxins produced by *Clostridium* species [2]. BTX-A is used for cosmetic purpose in patients with masseter muscle hypertrophy [3]. In addition, BTX-A is used for reducing pain in temporomandibular disorders [4].

Tsai et al. reported that a decrease in muscle volume leads to a decrease in mandibular bone mass [5]. An experimental study on rabbits reported significant decrease in bone quality and quantity in the condylar head at 4 weeks after botulinum toxin injection (BTI). Although functional parameters reverted to near pre-injection level at 12 weeks after BTI, the muscle (masseter) remained atrophic and percentage of bone area in the condylar head was low [6].

Bisphosphonates (BPs) suppress bone resorption through various mechanisms such as decrease in turnover rate, inhibition of activity, and induction of apoptosis of osteoclasts [7, 8]. In this regard, BPs have been used for the prevention of osteoporosis, bone metastasis of malignant tumor, and pathologic fracture.

Thus, we hypothesized that the application of bisphosphonate after BTX-A (injected to unilateral masticatory muscles) could contribute to the inhibition of bone resorption or osteopenic condition of ipsilateral condylar through inhibition of osteoclast action. The purpose of this study was to evaluate the effect of bisphosphonate administration on the prevention of condylar resorption and botulinum toxin A-induced disuse osteopenia in rats.

## Methods

### Animal model and study design (Fig. 1)

Sixteen female Sprague-Dawley (SD) rats (Orientbio Co. Ltd., Seongnam-si, Gyenggi-do, Korea) were used in the present study. All rats were 10-week-old of age. Two rats were housed per cage and were individually marked. The cages were placed in a room with filtered air at a temperature of 22 °C ± 2 °C and 50% ± 10% relative humidity. A 12-h light/dark cycle was maintained. The animals were fed a normal rodent diet and water ad libitum. Animals were acclimated for 1 week prior to the beginning of the study. This study was approved by and performed in accordance with the guidelines of the institutional animal research ethics committee (IACUC No. 2017-0083).

**Fig. 1:**
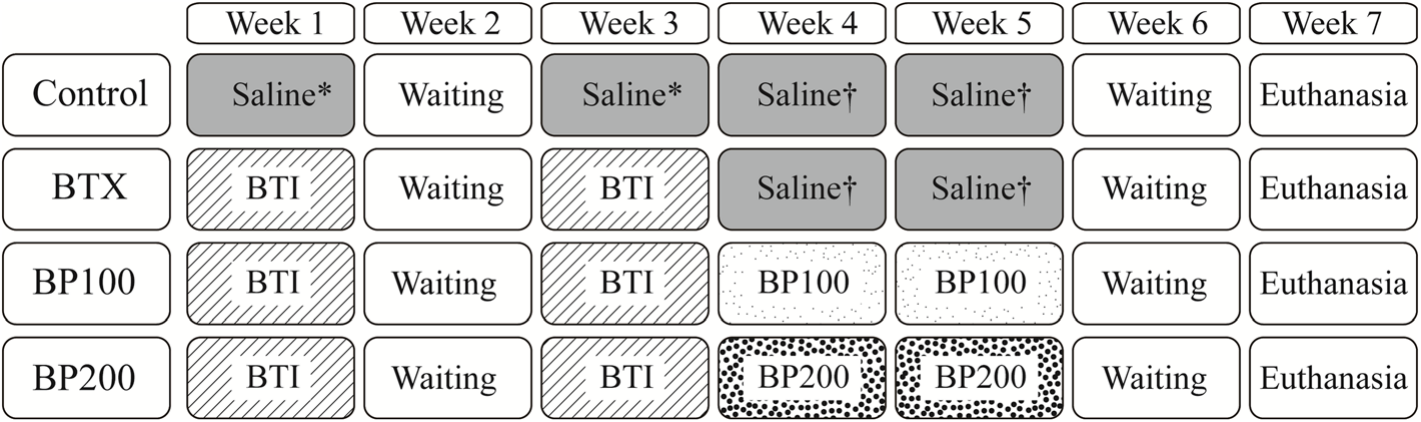
Experiment design. 3.75 U of botulinum toxin and the same dose of normal saline were administered in weeks 1 and 3. In weeks 4 and 5, 100 μg/kg or 200 μg/kg of bisphosphonate (zoledronic acid) was administered to the respective groups. A total of 100 μg/kg of normal saline was administered to both the control and BTX groups. All animals were euthanized on week 7

The animals were randomly divided into four groups: control, Botox (BTX), bisphosphonate 100 (BP100), and bisphosphonate 200 (BP200) (*n* = 4). In the first and third week, 3.75 U of botulinum toxin injection (BTI) (Botulax®, Botulinum toxin A, HUGEL, Chuncheon, Korea) was administered on the left side of the masseter (2.5 U) and temporalis muscles (1.25 U) in all groups except the control group under general anesthesia with isoflurane. In the control group, same dosage of normal saline was administered on the left side of the masseter and temporalis muscles.

A total of 100 μg/kg (for BP100 group) or 200 μg/kg (for BP200 group) of zoledronic acid (ZA, Zometa ready®, Novartis, Switzerland) was injected into the peritoneum, for 2 weeks after injection of BTI (in fourth and fifth week of experiment). There was a 1-week waiting period (sixth week) for the effect of administered bisphosphonate. All the animals were euthanized when the seventh week of experiment began.

### Microscopic computed tomography

Microscopic computed tomographic (μCT, Skyscan 1173, Kontich, Belgium) images were scanned at 10.65-μm pixel size at an energy level of 90 kV and evaluated using CTAn® software (Skyscan, Kontich, Belgium).

**Fig. 2:**
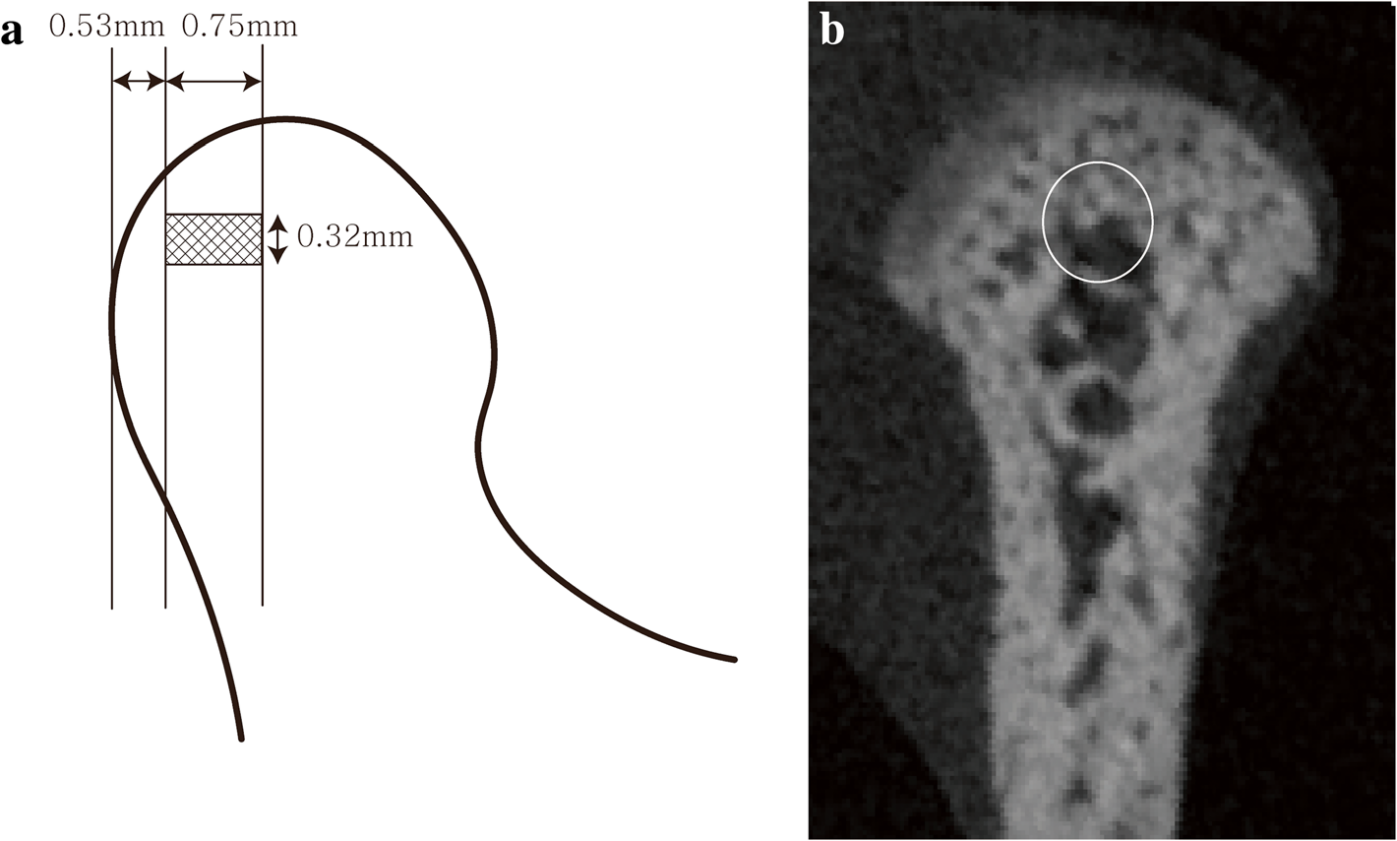
Range of analysis (**a**) and region of interest (ROI) (**b**). The region of interest (ROI) was focused between 0.53 and 1.28 mm from the most posterior margin of the condyle. Then, a 0.32 mm × 0.32 mm sized circle was set at the center of the condyle. Finally, cylindrical shape was formed at the condyle area (0.32 mm diameter and 0.75 mm height), and this area was analyzed

The region of interest (ROI) was focused between 0.53 mm and 1.28 mm from the most posterior margin of the condyle. Then, a 0.32 mm × 0.32-mm sized circle was set at the center of condyle. Finally, cylindrical shape was formed at the condyle area (0.32 mm diameter and 0.75 mm height), and this area was analyzed (Fig. 2a, b). Bone volume/tissue volume (BV/TV, mm^3^), trabecular numbers (Tb.N, 1/mm), trabecular thickness (Tb.Th, mm), and trabecular separation (Tb.Sp, mm) of each group were calculated by CTAn® software.

### Three-dimensional image reconstruction and analysis (Fig. 3)

μCT images were converted to the Digital Imaging and Communications in Medicine (DICOM) files using a converting software (DicomCT ver 2.0, Bruker®). The 3D images were then reconstructed and analyzed using image analysis software (Mimics Research 21.0, Materialise, Belgium). The threshold was set between − 500 and 750 Hounsfield Unit.

**Fig. 3:**
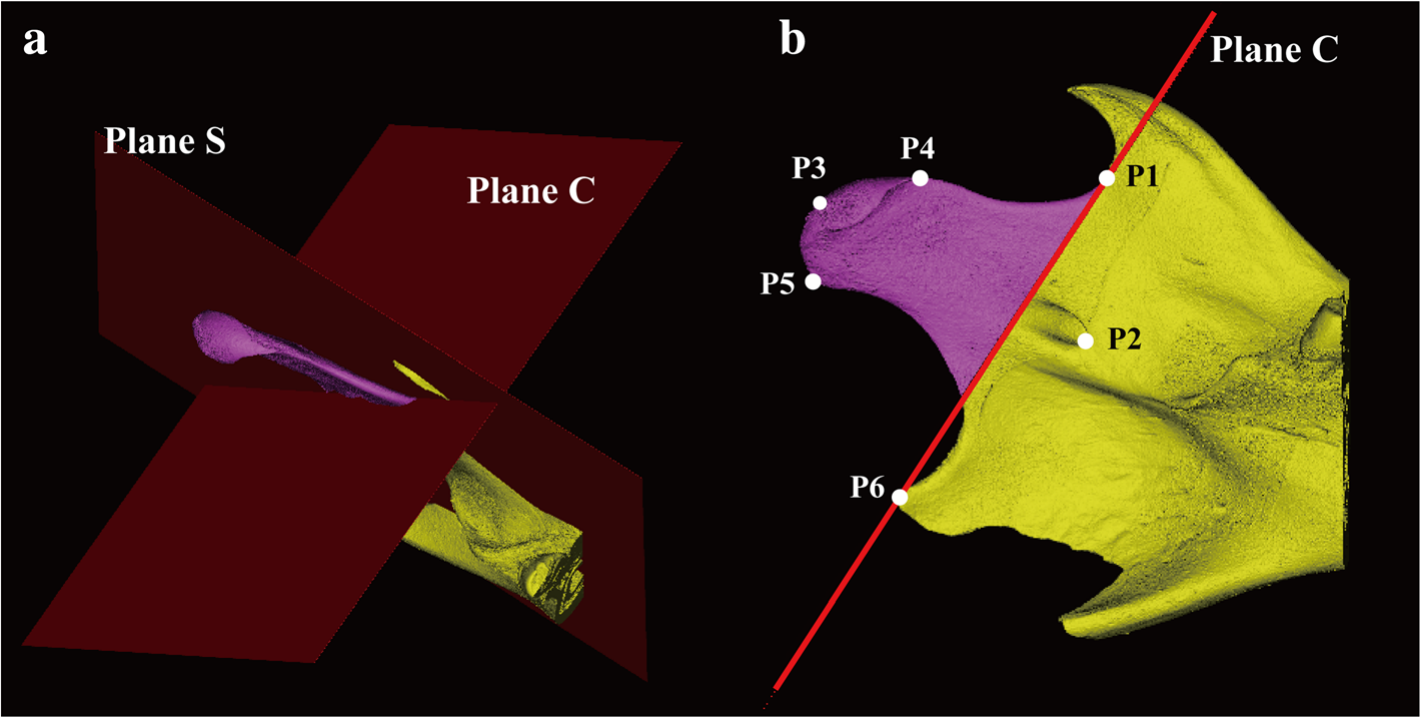
Reference planes (**a**) and reference points (**b**). Plane S refers to a plane passing through P1, P2, and P6. Plane C refers to a plane normal to plane S, passing through P1 and P6. The condyle component was separated by plane C from the other part of the mandible. P1, sigmoid notch; P2, mandibular foramen; P3, condyle superior; P4, condyle anterior; P5, condyle posterior; P6, angle

The reference points on the 3D images were defined as follows:

1. Sigmoid notch (P1)—the lowest point of the sigmoid notch.
2. Mandibular foramen (P2)—the most anterior point of the opening of the inferior alveolar nerve canal.
3. Condyle superior (P3)—the most superior point of the condyle.
4. Condyle anterior (P4)—the most anterior point of the condyle.
5. Condyle posterior (P5)—the most posterior point of the condyle.
6. Angle (P6)—the most prominent point of the mandibular angle.

The reference planes on the 3D images were defined as follows:

1. Sagittal plane (plane S)—a plane passing through P1, P2, and P6.
2. Coronal plane (plane C)—a plane normal to the plane S passing through P1 and P6.

The volume of condyle (V_Con) is measured above the plane S, which was calculated automatically by the analysis software (Fig. 3). Distance and angle from P1 and P2 to each point on the condyle (P3, P4, and P5) were measured, the values of which are summarized in Table 1.

**Table 1:** Definition of the volume of condyle angle and distance measurement

Measurements	Definition
V_Con (mm^3^)	Volume of condyle calculated above plane C
Ang123 (deg)	Angle between P1, P2, and P3
Ang124 (deg)	Angle between P1, P2, and P4
Ang125 (deg)	Angle between P1, P2, and P5
D12 (mm)	Distance between P1 and P2
D13 (mm)	Distance between P1 and P3
D14 (mm)	Distance between P1 and P4
D15 (mm)	Distance between P1 and P5
D23 (mm)	Distance between P2 and P3
D24 (mm)	Distance between P2 and P4
D25 (mm)	Distance between P2 and P5

*V_Con* volume of condyle, *Ang* angle, *D* distance, *Plane C* coronal plane, *P1* sigmoid notch, *P2* mandibular foramen, *P3* condyle superior, *P4* condyle anterior, *P5* condyle posterior, *P6* angle

### Statistical analyses

The data were analyzed using a statistical analysis program (SPSS 23.0, IBM, USA). μCT, the volume, and linear and angular values were compared using Kruskal-Wallis analysis. *p* values < 0.05 indicated as statistically significant.

## Results

One rat in the BTX and BP100 group died during the experiment. Therefore, the analysis was performed on three rats in the BTX and BP100 group and four rats in the other groups.

### μCT analysis (Table 2)

**Fig. 4:**
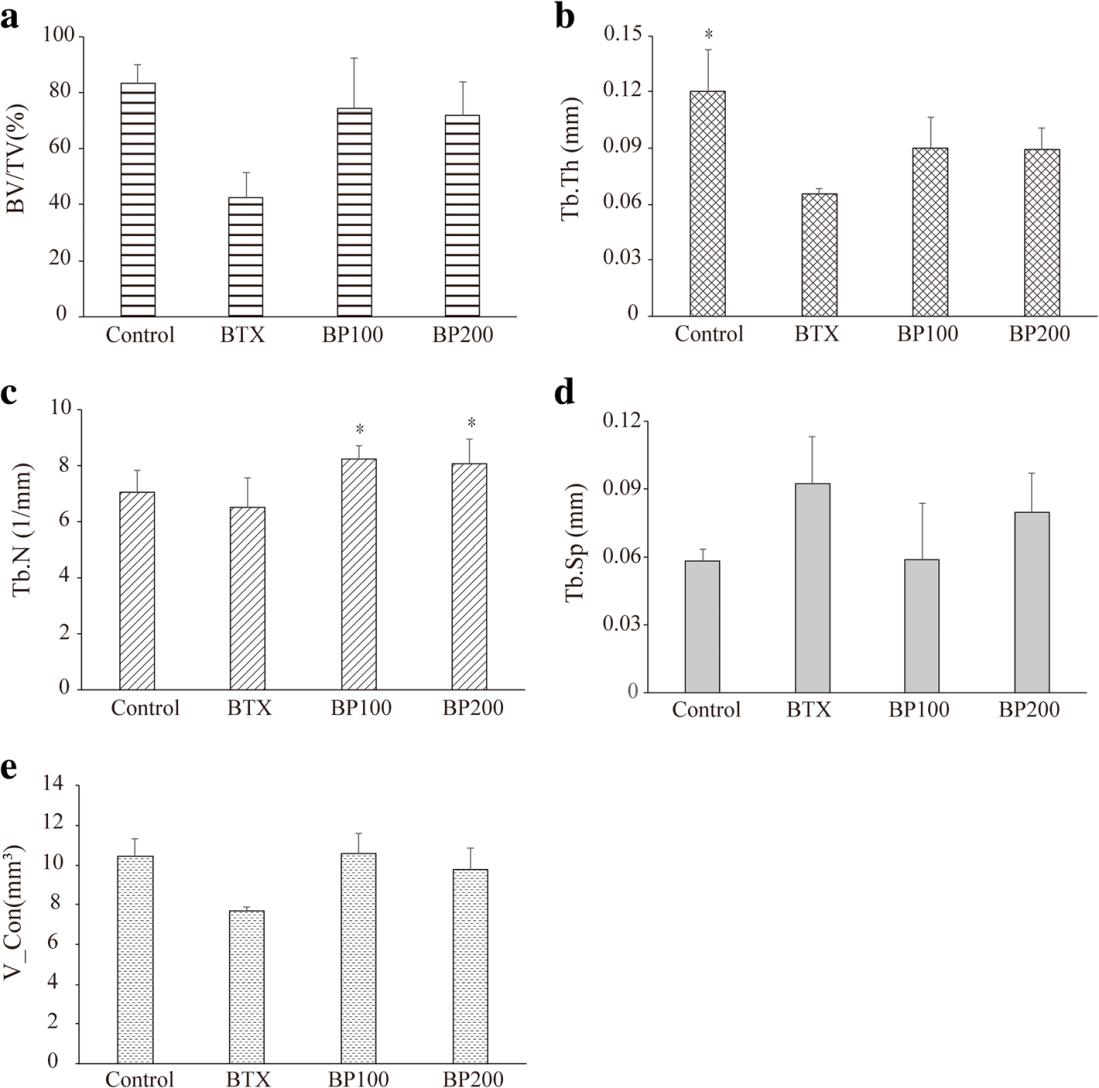
Microscopic computed tomography (μCT) analysis (**a**–**d**) and three-dimensional analysis of condylar volume (**e**). **a** Bone volume/tissue volume (BV/TV, %) in the BTX group was the lowest with marginal trend towards significance when compared to the other groups (*p* = 0.052). **b** Trabecular thickness (Tb.Th, mm) was the lowest in the BTX group (*p* = 0.025). However, significant difference was observed only between the BTX and control groups. **c** Trabecular number (Tb.N, 1/mm) was the lowest in BTX. BP100 and BP200 groups showed significant higher value compared with the BTX group. **d** Trabecular number (Tb.N, 1/mm) and trabecular separation (Tb.Sp, mm) showed no significant difference among the four groups. **e** Volume of the condyle on three-dimensional analysis showed similar result with BV/TV (%). *Statistically significant difference compared with the BTX group (*p* < 0.05)

The BTX group had the lowest BV/TV (%), with marginal trend towards significance when compared to the other groups (*p* = 0.052). However, other groups showed similar values. Tb.Th (mm) and Tb.N (1/mm) values were the lowest in the BTX group with statistically significant difference (*p* = 0.025 and *p* = 0.044, respectively). Tb.Sp (mm) was the highest in the BTX group. However, no significant difference was observed among the four groups (Fig. 4a–d).

**Table 2:** Microscopic computed tomography (μCT) analysis of the left condyle

	Control (*n* = 4)	BTX (*n* = 3)	BP100 (*n* = 3)	BP200 (*n* = 4)	*p* value
BV/TV (%)	83.41 ± 6.69	42.55 ± 8.94	74.66 ± 17.66	72.23 ± 11.87	0.052
Tb.Th (mm)*	0.12 ± 0.02	0.07 ± 0.00	0.09 ± 0.02	0.09 ± 0.01	0.025
Tb.N (1/mm)*	7.03 ± 0.79	6.50 ± 1.05	8.22 ± 0.47	8.07 ± 0.87	0.044
Tb.Sp (mm)	0.06 ± 0.01	0.09 ± 0.02	0.06 ± 0.02	0.08 ± 0.18	0.102

*BTX* botulinum toxin, *BP100* 100 μg/kg of bisphosphonate, *BP200* 200 μg/kg of bisphosphonate, *BV/TV* bone volume/tissue volume, *Tb.Th* trabecular thickness, *Tb.N* trabecular number, *Tb.Sp* trabecular separation

*Significant difference (*p* < 0.05), mean ± SD

### Volumetric, linear, and angular measurement (Table 3)

The volume of the condyle was the smallest in the BTX group (7.68 ± 0.19 mm^3^), with at the edge of significance when compared to the other groups (*p* = 0.058). The condylar volumes were 10.44 ± 0.89 mm^3^, 10.58 ± 1.02 mm^3^, and 9.81 ± 1.03 mm^3^ in the control, BP100, and BP200 groups, respectively (Fig. 4e).

**Table 3:** Volumetric, angular, and linear analysis of the left side of the mandible

	Control (*n* = 4)	BTX (*n* = 3)	BP100 (*n* = 3)	BP200 (*n* = 4)	*p* value
V_Con (mm^3^)	10.44 ± 0.89	7.68 ± 0.19	10.58 ± 1.02	9.81 ± 1.03	0.058
Ang123 (deg)	63.14 ± 3.33	70.72 ± 5.07	67.86 ± 5.29	61.87 ± 4.57	0.123
Ang124 (deg)	43.57 ± 5.18	56.79 ± 6.80	53.01 ± 4.41	47.09 ± 4.82	0.080
Ang125 (deg)	80.93 ± 5.62	84.71 ± 6.31	87.23 ± 6.00	80.48 ± 4.26	0.473
D12 (mm)	3.98 ± 0.08	3.86 ± 0.33	3.67 ± 0.30	3.83 ± 0.17	0.454
D13 (mm)	6.57 ± 0.07	7.10 ± 0.54	7.00 ± 0.30	6.58 ± 0.23	0.073
D14 (mm)*	3.66 ± 0.40	5.11 ± 0.57	4.74 ± 0.22	4.34 ± 0.32	0.014
D15 (mm)	7.14 ± 0.18	7.72 ± 0.48	7.56 ± 0.37	7.33 ± 0.35	0.158
D23 (mm)	7.33 ± 0.31	7.34 ± 0.24	7.49 ± 0.12	7.44 ± 0.10	0.564
D24 (mm)*	5.30 ± 0.21	6.04 ± 0.12	5.92 ± 0.18	5.92 ± 0.07	0.026
D25 (mm)	6.60 ± 0.29	7.03 ± 0.10	6.78 ± 0.09	6.92 ± 0.21	0.064

*BTX* botulinum toxin, *BP100* 100 μg/kg of bisphosphonate, *BP200* 200 μg/kg of bisphosphonate, *V_Con* volume of condyle, *Ang* angle, *D* distance

*Significant difference (*p* < 0.05), mean ± SD

Among D13, D14, D15, D23, D24, and D25 (the distance from P1 (sigmoid notch) or P2 (lingula) to P3 (condyle superior), P4 (condyle anterior), and P5 (condyle posterior)), only D14 and D24 showed a significant difference (*p* < 0.05).

The angle between P1, P2, and P4 (Ang124) was the largest in the BTX group (56.79 ± 6.80°) and the smallest (43.57 ± 5.18°) in the control group (*p* = 0.080).

## Discussion

Botulinum toxin A (BTX-A) has been used for the treatment of masseter hypertrophy, masticatory myalgia, and temporomandibular joint disorders [3, 4, 9]. Injection of BTX-A into the masticatory muscles induces transient muscle paralysis, which may increase the risk of reduction in bone mineral density, or “disuse atrophy” [10]. Several studies have reported on the resorption and/or trabecular changes in the condyle [5, 9–11]. These changes may occur in both experimental conditions and clinical situations. In the present study, 3.75 U of botulinum toxin A was injected in the left masseter muscle (2.5 U) and left temporal muscle (1.25 U) under general anesthesia with isoflurane based on the protocol reported in previous studies [9, 12].

Bisphosphonates suppress osteoclastic activity and induce apoptosis of osteoclasts, thereby exerting a protective effect on bone loss [7, 8, 13]. Several investigators reported that BPs prevented subchondral bone loss on articulation in experimental animal studies [14–16]. Chen et al. specifically reported that the injection of BP into the temporomandibular joint articulation could reduce changes in cartilage and subchondral bone loss [16].

In this regard, the hypothesis of the present study was that the administration of BP after botulinum toxin A injection into masticatory muscles has a protective potential in the maintenance of the volume of the condyle.

One study reported that the dose of bisphosphonate (zoledronate) administered to prevent bone metastasis in a patient with malignant tumor was 67 μg/kg every 4 weeks [17]. Black et al. reported an increase in bone mineral density on yearly infusion of 5 mg zoledronic acid by intravenous route [18]. The dose amounts to approximately 7 μg/kg/week, when converted, in a 60 kg adult. In a study by Chen et al., which investigated the prevention of condyle resorption in rats, 100 μg of alendronate was administered at weekly intervals for 4 weeks [16]. In the present study, 100 μg/kg (BP100 group) and 200 μg/kg (BP200 group) bisphosphonate were administered considering (1) relatively fast bone turnover rate in rats, (2) short-term dosing period (2 weeks), and (3) intraperitoneal route of administration.

As a result, the volume of the condyle showed similar values between the BP100 group (receiving 100 μg/kg) and the BP200 group (receiving 200 μg/kg). The volume of the condyle was the lowest in the BTX group with a strong tendency towards statistical significance when compared with other groups (*p* = 0.058). In the μCT analysis, the BV/TV of BTX group also showed the lowest value with a strong tendency towards statistical significance compared with the other three groups (*p* = 0.052).

Both cortical bone thickness and trabecular bone area decreased after the injection of botulinum toxin into the masticatory muscles [1, 5]. Kün-Darbois et al. also reported a significant decrease in bone area/tissue area (B.Ar/T.Ar) on trabecular bone after the injection of botulinum toxin compared with the control group in μCT analysis [9]. Recently, Vegger et al. reported that injection of 100 μg/kg zoledronic acid via the subcutaneous route prevented disuse osteopenia induced by botulinum toxin. Although the study focused on the limb and not the condyle, our group hypothesizes a similar effect on the temporomandibular joint [19].

Further studies with larger sample sizes are required to establish the preventive effect of bisphosphonate on disuse osteopenia and condylar resorption.

## Conclusion

The result of the present study suggests that bisphosphonate administration after botulinum toxin A injection into the masticatory muscle may have a preventive effect on condyle resorption and botulinum toxin-induced disuse osteopenia in rats. However, the finding of this study is preliminary and requires further evaluation.

## Abbreviations

B.Ar/T.Ar: Bone area/tissue area
BP: Bisphosphonate
BTI: Botulinum toxin injection
BTX-A: Botulinum toxin A
BV/TV: Bone volume/tissue volume
DICOM: Digital Imaging and Communications in Medicine
ROI: Region of interest
Tb.N: Trabecular numbers
Tb.Sp: Trabecular separation
Tb.Th: Trabecular thickness
μCT: Microscopic computed tomography
